# Electrochemotherapy and Calcium Electroporation on Hepatocellular Carcinoma Cells: An In-Vitro Investigation

**DOI:** 10.1007/s00270-024-03847-1

**Published:** 2024-09-03

**Authors:** K. H. K. Lindelauf, M. Baragona, T. Lemainque, R. T. H. Maessen, A. Ritter

**Affiliations:** 1 Department of Diagnostic and Interventional Radiology, University Hospital RWTH Aachen, Aachen, Germany; 2 Philips Research, Eindhoven, The Netherlands

**Keywords:** Electrochemotherapy, Calcium electroporation, ESOPE, Hepatic cancer, HepG2, In vitro

## Abstract

**Purpose:**

Electrochemotherapy, clinically established for treating (sub)cutaneous tumors, has been standardized in the framework of the European Standard Operating Procedure on Electrochemotherapy (ESOPE). Due to common side effects of chemotherapeutic drugs, recent advances focus on non-cytotoxic agents, like calcium, to induce cell death (calcium electroporation). Therefore, this study aims to determine the efficacy of electrochemotherapy with bleomycin or cisplatin, or calcium electroporation on human hepatocellular carcinoma cells (HepG2) in vitro using the ESOPE protocol.

**Methods:**

HepG2 cell viability was measured with a MTT (3-(4,5-dimethylthiazol-2-yl)-2,5-diphenyltetrazolium bromide) assay after electrochemotherapy with the chemotherapeutic drugs bleomycin or cisplatin (0–20 µM), or after calcium electroporation (0–20 mM), to determine its efficacy on HepG2 cells in vitro using the ESOPE protocol (8 rectangular pulses, 1000 V/cm, 100 µs) compared to non-electroporated drug treatment.

**Results:**

Cell viability was significantly lower in electroporated samples, compared to their non-electroporated controls (27–75% difference). Electrochemotherapy with bleomycin and calcium electroporation, reached (almost) complete cell death (− 1 ± 3% and 2.5 ± 2%), in the lowest concentration of 2.5 µM and 2.5 mM, respectively. Electrochemotherapy with 2.5 µM cisplatin, significantly decreased cell viability to only 68% (± 7%).

**Conclusion:**

Electrochemotherapy with bleomycin or cisplatin, or calcium electroporation were more effective in reducing the HepG2 cell viability in vitro using the ESOPE protocol compared to the non-electroporated drug treatments alone. When comparing electrochemotherapy, HepG2 cells are more sensitive to bleomycin than cisplatin, in similar concentrations. Calcium electroporation has the same effectiveness as electrochemotherapy with bleomycin, but calcium potentially has a better safety profile and several treatment advantages.

**Graphical Abstract:**

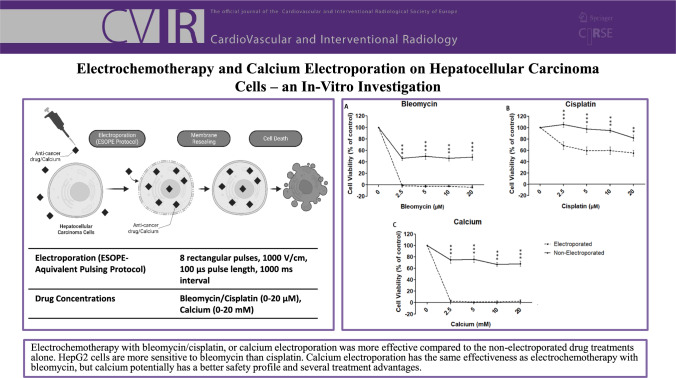

## Introduction

Cancer is the second leading cause of death worldwide, and the number of patients is still rising rapidly in aging populations [1]. Hepatocellular carcinoma (HCC), accounting for > 80% of primary liver cancers, has a heavy disease burden and is a leading cause of cancer-related death worldwide [2, 3]. The liver is also a frequent location of metastases for various tumor diseases [4, 5]. Traditional treatment options are not always effective. As with any cancer, the treatment and prognosis of HCC varies depending on the specifics of tumor histology, size, metastatic status, and the overall health of the patient. [6]

Over the past years, minimally invasive cancer therapies have been an important area of research, with nonthermal electroporation (EP)-based therapies among the most promising approaches, as it provides the possibility to operate in proximity to vulnerable structures [7]. In EP-based therapies, a sufficiently high pulsed electric field (PEF) is applied to induce permeabilization of the cell membrane, creating so-called nanopores. [7, 8]

**Fig. 1:**
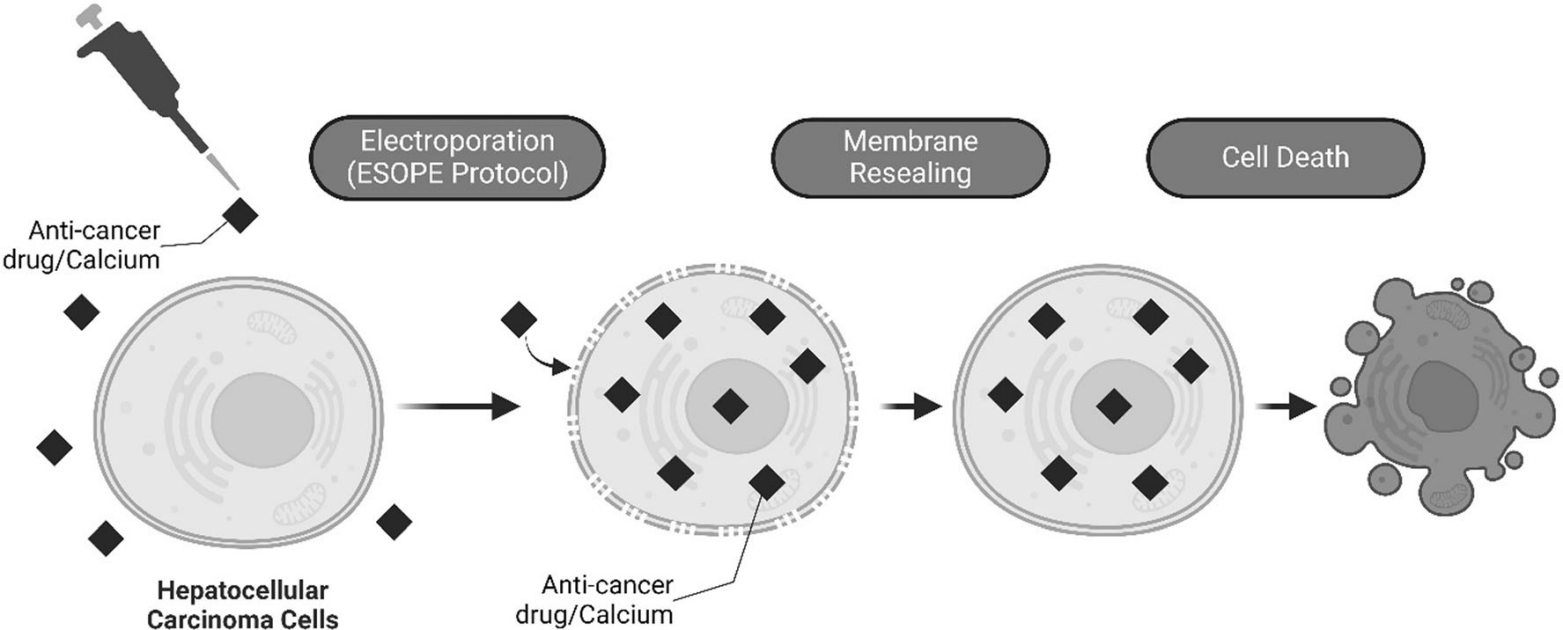
Principles of electrochemotherapy (ECT) and calcium electroporation (CaEP)

During irreversible electroporation (IRE), these nanopores cannot be repaired because of their size and amount which leads to cell death [7]. During reversible electroporation (RE), the cell can repair its membrane and continue normal cell function. In clinical practice, those transient hydrophilic pores are used to promote the diffusion of hydrophilic chemotherapeutic drugs, most commonly bleomycin or cisplatin. This therapeutic approach is known as Electrochemotherapy (ECT) (Fig. 1) [8, 9]. Currently, ECT is an established and efficient option in the clinic for the treatment of (sub)cutaneous tumors [10]. Its treatment protocol has been standardized in the framework of the European Standard Operating Procedure on ECT (ESOPE). [11, 12] Recent advances focus on the development of ECT for deep-seated tumors, such as liver cancer. Although the first clinical studies showed promising results, the intraoperative procedure during open surgery was considered a limitation [13]. This limitation has recently been overcome with the development of a new pulse generator Cliniporator^®^VITAE (IGEA SpA, Carpi, Italy) in combination with long needle electrodes, which can generate sufficient power to treat deep-seated tumors percutaneously. The first attempts on HCC patients have shown this method to be safe, feasible and efficient. [14, 15]

Due to common side effects of chemotherapeutic drugs, recent advances focus on the investigation of non-cytotoxic agents, such as calcium (as compound often administered as the salt calcium chloride), which can also be internalized into the cells in high concentration by electroporation to induce cell death (Fig. 1) [16, 17]. Calcium is a tightly regulated ubiquitous intracellular second messenger involved in many cellular processes, including cell death [18]. Calcium electroporation (CaEP) has shown to efficiently induce cell death in vitro, in vivo, and in clinical trials, through adenosine triphosphate (ATP) depletion, using electroporation parameters similar to the ESOPE protocol for ECT [19–22]. In a previous study, we have verified the ESOPE protocol for RE on human HCC cells (HepG2) in vitro*,* but without performing ECT or CaEP yet, i.e., without the application of chemotherapeutic drugs or calcium. [23]

Based on these promising results, more basic research needs to be done to transfer CaEP to clinical practice, and to extend the application of ECT to deep-seated tumors like liver cancer.

Therefore, this follow-up study aims to determine the efficacy of ECT with bleomycin or cisplatin, or CaEP on HepG2 cells in vitro using the ESOPE protocol, compared to non-electroporated drug treatment.

## Methods

In this translational study, the HepG2 cell viability was measured after ECT with the chemotherapeutic drugs bleomycin or cisplatin, or after CaEP, to determine its efficacy on HepG2 cells in vitro using the ESOPE protocol, compared to non-electroporated drug treatment.

### Cell Culture

The human HCC cell line HepG2 (ATCC, Manassas, US), derived from a Caucasian male, was used for all in vitro experiments. As described previously, the cells were routinely subcultured twice a week (1:5 ratio) by trypsinization. Eagle’s Minimum Essential Medium (EMEM) (ATCC, Manassas, US) supplemented with 1% penicillin/streptomycin (100 U/mL and 100 µg/mL) and 10% fetal bovine serum (FBS) (100 µL/mL) was used as culture medium. The cells were grown in a humidified incubator (37 °C with 5% CO2). [23]

### Electroporation Parameters

The RE electroporation parameters for HepG2 cells were determined and verified in a previously conducted study, where a detailed description of the materials and methods can be found. [23]

In an in vitro setup, HepG2 cell viability was measured with a Trypan Blue dye exclusion assay at 0, 5, 10 and 15 min after electroporation with a RE pulsing protocol (8 rectangular pulses, 100 µs pulse length, 1000 ms interval resp. 1 Hz) combined with variable electric field strengths (0–4000 V/cm), to determine the most successful settings for RE (n = 9, in duplicate). To confirm cell permeabilization for two selected RE pulsing protocols (500 and 1000 V/cm), a Calcein acetoxymethyl ester (CAM)/Propidium Iodine (PI) flow cytometric assay was performed (n = 3, in duplicate).

### Electrochemotherapy and Calcium Electroporation

A suspension of 1 million HepG2 cells/mL was prepared in a HEPES-based low-conductivity electroporation buffer (HEPES 10 mM, Sucrose 250 mM, MgCL_2_ 1 mM). A variety of cell-drug suspensions was prepared. The final drug concentration was varied between 0 (control), 2.5, 5, 10 or 20 µM bleomycin (BLEO-cell, STADAPHARM GmbH, Bad Vilbel, DE) or cisplatin (Cisplatin Teva, TEVA GmbH, Ulm, DE), and 0 (control), 2.5, 5, 10 or 20 mM calcium (calcium chloride dihydrate, CaCl_2_·2H_2_O, Sigma-Aldrich, St. Louis, USA). 400 µL of the abovementioned cell-drug suspensions was added to a BTX Electroporation Cuvette Plus (2 mm, 400 µL) (BTX, Holliston, US), powered by a Gemini Twin Wave Electroporator (BTX, Holliston, US). The samples were either electroporated following the ESOPE protocol (8 rectangular pulses, 1000 V/cm, 100 µs pulse length, 1000 ms interval resp. 1 Hz), previously confirmed for this cell type, or sham-exposed and non-electroporated [23]. The sample size was based on preliminary data, and consisted of 9 independent experiments, measured in duplicates (n = 9). [23]

The samples were centrifuged (5000 rpm, 5 min) and the supernatant was discarded. The samples were resuspended in culturing medium and seeded (10.000 cells/well, in duplicates) in a 96 well plate (VWR, Radnor, US). A blank well with only culturing medium served as background control.

After the plates were incubated for 72 h at 37 °C, a MTT ((3-(4,5-dimethylthiazol-2-yl)-2,5 diphenyl tetrazolium bromide) assay (Thermo Fisher Scientific, Waltham, US) was performed to measure the HepG2 cell viability. The medium was replaced with 100 µL of fresh medium, and 10 µl of 12 mM MTT solution was added to each well. The plates were incubated for 4 h at 37 °C. After the incubation, 25 µl medium was kept in the wells and 50 µl DMSO (AppliChem GmbH, Darmstadt, DE) was added and mixed thoroughly. After incubation for 10 min at 37 °C the samples were mixed again, and the absorbance was read at 540 nm using a BioTek Synergy HT Microplate Reader and Gen5 microplate reader software (BioTek Instruments, Winooski, US).

#### Statistics

The collected data was analyzed. For all samples, the duplicates were averaged, the background absorbance was subtracted, and the cell viability was calculated. Control groups without drug were set to 100% viability. Accordingly, the cell viability of the experimental samples was normalized to the corresponding control group and visualized using GraphPad Prism 5 (GraphPad Software, San Diego, US). Obvious outliers have been removed from the data set. Results were displayed in graphs as mean ± standard error of the mean (SEM). A two-way analysis of variance (ANOVA), with Bonferroni-correction as pair-wise post-hoc comparison, was performed between corresponding electroporated and non-electroporated samples (visually indicated in Fig. 3), and between the different drug concentrations (not visually indicated in Fig. 3, to avoid confusion). two-tailed p-values of *p* < 0.05 were interpreted as statistically significant (*).

## Results

### Electroporation parameters

**Fig. 2:**
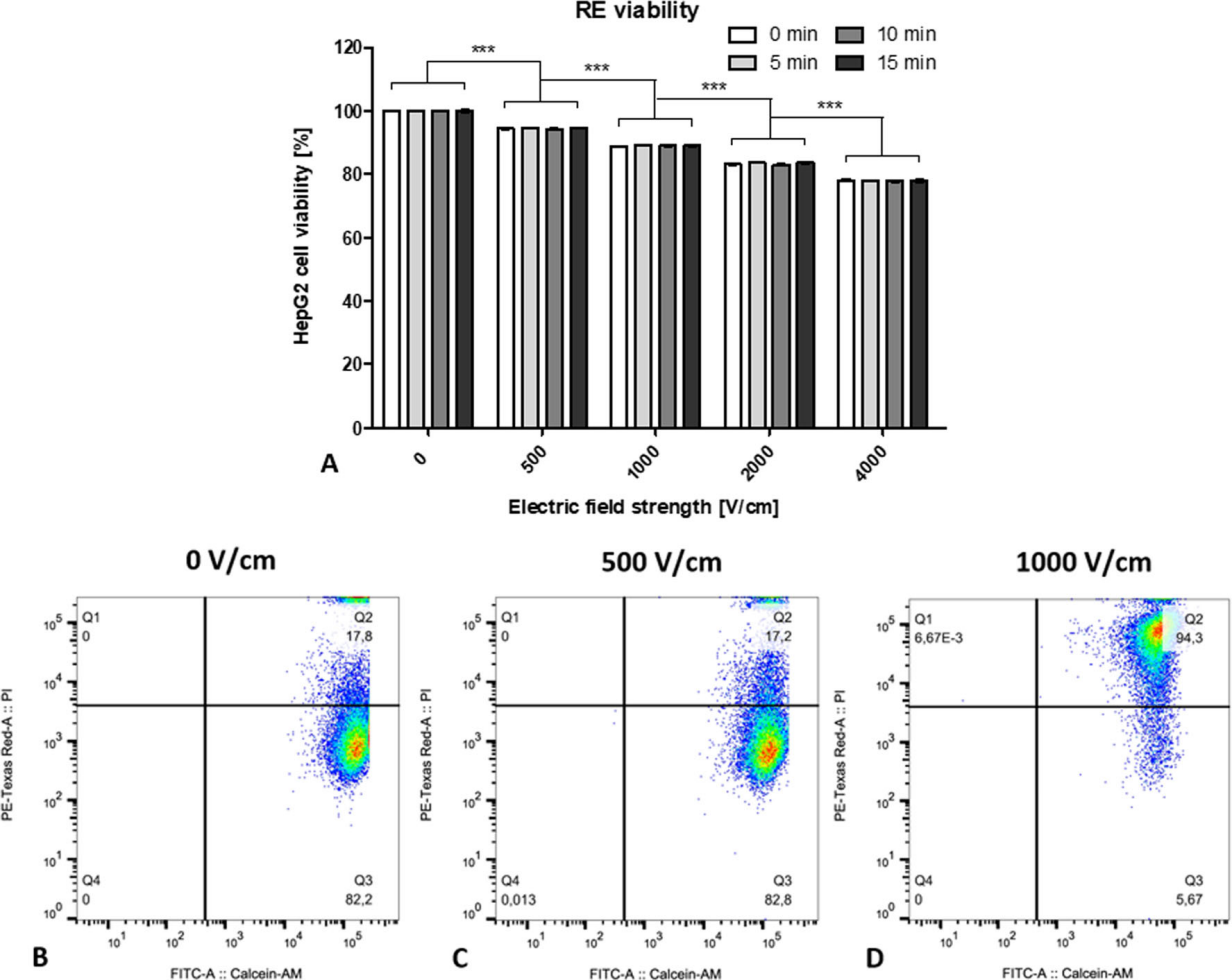
**A** Cell viability (% of control) of HepG2 cells at 0, 5, 10 and 15 min after electroporation with a RE pulsing protocol (8 pulses, 100 µs pulse length) combined with variable electric field strengths (0–4000 V/cm), measured with a Trypan Blue dye exclusion assay. The results are displayed as mean ± SD. A two-way ANOVA with Bonferroni correction was performed between the different field strengths (results of pair-wise post hoc comparisons indicated above the data points, *p < 0.05, **p < 0 .01, ***p < 0.001). n = 9, in duplicates. **B** Representative dot-plot graph from CAM/PI flow cytometric assay on HepG2 cells. RE pulsing protocol with an electric field strength of 0 V/cm (**B**), 500 V/cm (**C**), and 1000 V/cm (**D**). The number of cells is represented by the color intensity. Cells were characterized as viable and nonpermeabilized (Q3; CAM+/PI−), viable and permeabilized (Q2; CAM+/PI+), dead (Q1; CAM−/PI+), and unstained debris (Q4; CAM−/PI−). n = 3, in duplicates.^23^

A RE pulsing protocol (8 rectangular pulses, 100 µs pulse length, 1000 ms interval resp. 1 Hz) with an electric field strength of 1000 V/cm was needed as threshold for viable and permeabilized HepG2 cells (Fig. 2). These parameters correspond to the ESOPE protocol. [23]

### Electrochemotherapy and Calcium Electroporation

The HepG2 cell viability was measured and compared after ECT with increasing concentrations of the chemotherapeutic drugs bleomycin or cisplatin, or after CaEP following the ESOPE pulsing protocol.

**Fig. 3:**
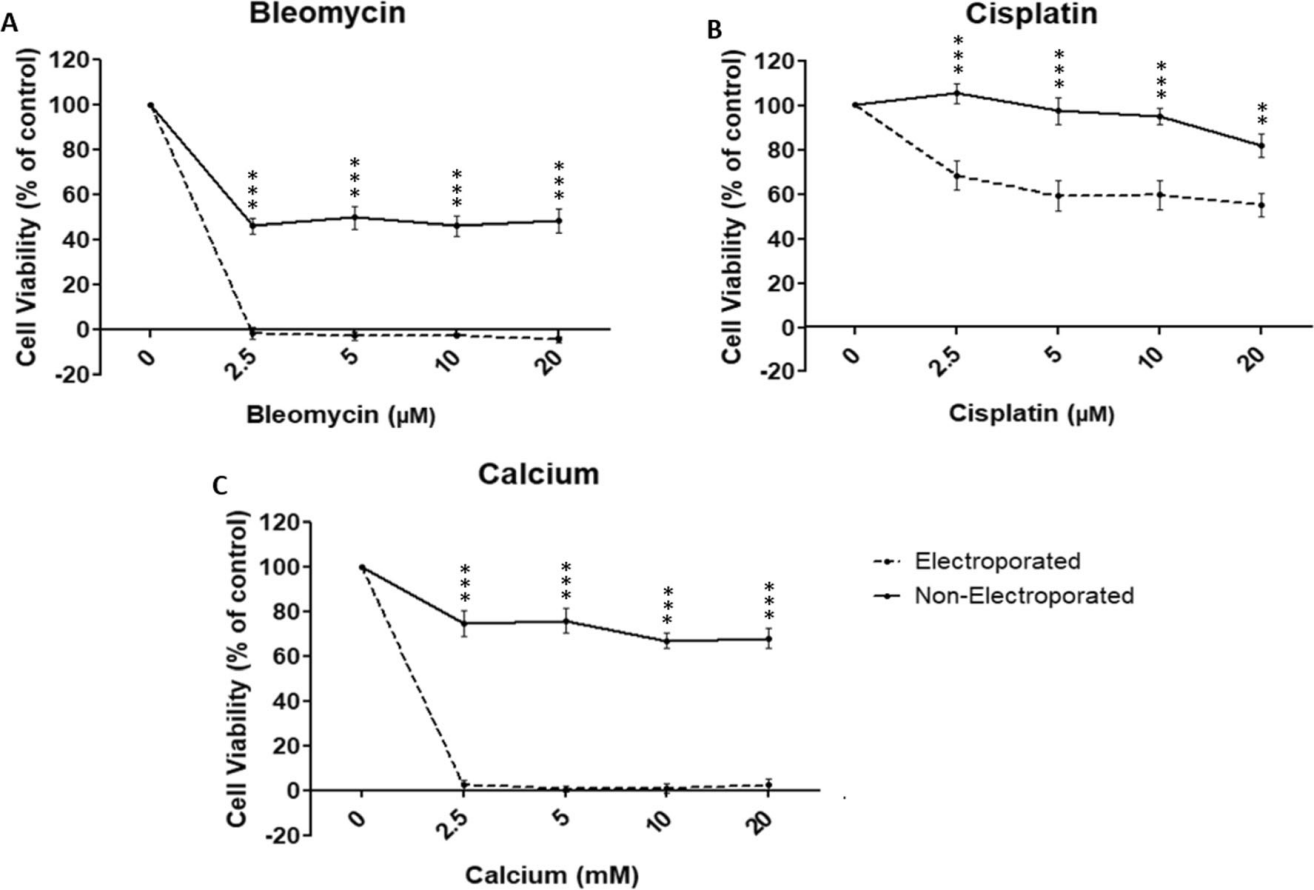
Cell Viability (% of control) of HepG2 cells after ECT with the chemotherapeutic drugs bleomycin (**A**) or cisplatin (**B**), or after calcium-electroporation (**C**) with the ESOPE pulsing protocol (8 pulses, 1000 V/cm, 100 µs), measured with an MTT assay. Sham-exposed non-electroporated samples served as control. Results are displayed as mean ± SEM. A two-way ANOVA with Bonferroni correction was performed between corresponding electroporated and non-electroporated samples (results of pair-wise post hoc comparisons indicated above the data points, *p < 0.05, **p < 0 .01, ***p < 0.001), and between the different drug concentrations (post hoc comparison results not visually indicated). n = 9, in duplicates

For all administered concentrations of bleomycin, cisplatin, or calcium, the HepG2 cell viability was significantly lower in the electroporated sample, compared to its non-electroporated control (*p* < 0.001) (Fig. 3).

After ECT with 2.5 µM bleomycin, the cell viability significantly decreased to -1% (± 3%), indicating complete cell death (*p* < 0.001). After administration of 2.5 µM bleomycin alone, without electroporation, the cell viability significantly decreased, but only to 46% (± 4%) (p < 0.001). For both ECT and the non-electroporated treatment, the further increase of bleomycin concentration to 5, 10, and 20 µM, did not result in a significant change in HepG2 cell viability (*p* > 0.05) (Fig. 3A).

After ECT with 2.5 µM cisplatin, the cell viability significantly decreased to 68% (± 7%), whereas 20 µM Cisplatin was needed to significantly decrease the cell viability to 82% (± 5%) in the non-electroporated sample (*p* < 0.001). Within the ECT treatment, the further increase of cisplatin concentration to 5, 10, and 20 µM, did not result in a significant change in HepG2 cell viability (*p* > 0.05) (Fig. 3B).

After CaEP with 2.5 mM calcium, the cell viability significantly decreased to 2.5% (± 2%), indicating almost complete cell death (*p* < 0.001). After administration of 2.5 mM calcium alone, without electroporation, the cell viability significantly decreased, but only to 75% (± 6%) (*p* < 0.001). For both CaEP and the non-electroporated treatment, the further increase of calcium concentration to 5, 10, and 20 mM, did not result in a significant change in HepG2 cell viability (*p* > 0.05) (Fig. 3C).

## Discussion

In this in vitro study, the HepG2 cell viability was measured after ECT with increasing concentrations of the chemotherapeutic drugs bleomycin or cisplatin, or after CaEP, to determine its efficacy on HepG2 cells in vitro using the ESOPE protocol, compared to non-electroporated drug treatment.

The colorimetric MTT assay, which assesses cell metabolic activity, was used as a representation of HepG2 cell viability [19]. One might argue that relatively high concentrations of bleomycin are used in this in vitro study. However, the concentration range for the drug bleomycin, as well as cisplatin and calcium was chosen to include the dosage used in literature for relevant and comparable in vitro studies, with proven effectiveness [12, 19, 21, 24–26]. These studies selected 10 µM bleomycin as optimal in vitro concentration from a test range of 0.1–50 µM bleomycin [21, 24]. In addition, the goal of this paper was not to find the optimal dosage for the drugs, but to confirm the effectiveness of electroporation combined with classic chemotherapeutic drugs, and CaEP as a novel addition, on HepG2 cells. By using similar literature, and adding a few higher and lower concentrations, the test range of the dosage needed for an effective treatment could be limited.

For all administered concentrations of bleomycin, cisplatin, or calcium, the combination with electroporation was more effective (minimum 27% and maximum 75% difference) in reducing the HepG2 cell viability compared to the non-electroporated controls, emphasizing the overall potential for ECT and CaEP. In general, to treat tumors with the same efficiency, ECT and CaEP use less drugs than conventional chemotherapies. This is in line with literature, as electro-permeabilization studies with classic chemotherapeutics have reported an enhanced cytotoxicity up to several thousand-fold for bleomycin, and up to 80-fold for cisplatin due to an increased cellular uptake and accumulation of the drug [27, 28]. Moreover, our results also show that, within the same concentration range, ECT with bleomycin is more effective than with cisplatin to reduce HepG2 cell viability. Thus, supporting the role of bleomycin as the chemotherapeutic drug of choice for traditional ECT.

ECT with bleomycin and CaEP had a similar effect on HepG2 cell viability, reaching (almost) complete cell death in the lowest concentration of 2.5 µM and 2.5 mM, respectively. Further concentration increase of the drugs did not cause any significant changes. However, it has been reported that 10 µM bleomycin and 5 mM calcium are considered to be the optimal concentrations in vitro as they cause 80% cell death [19, 21, 24, 25]. This suggests that the HepG2 cells within our study were more sensitive to both drugs in combination with electroporation. This underlines that more research is needed to optimize the drug concentrations for different cell types. It has been reported that several physical and biological cell properties can affect electroporation efficiency. [12, 29]

Despite their equal effectiveness combined with electroporation, bleomycin and calcium display different cytotoxic profiles when used as drug alone without electroporation. Calcium alone caused a reduction of HepG2 cell viability to 75%, compared to 46% for bleomycin, this suggests that CaEP is an equally effective but safer option. Similar studies, for different cell lines, have even reported that calcium alone had no significant impact on cell viability in comparable concentrations up to 20 mM. [12, 19, 30] Initial clinical studies have also found high doses of calcium (220–225 mM) to be well tolerated, contrary to classic chemotherapeutics. [22, 31]

Besides calcium’s potentially good safety profile, especially for patients, but also for the staff handling the drug, it is efficient, easily available, inexpensive, heat-stable and has a long shelf life [32]. This shows that CaEP, compared to traditional ECT, has the potential to be an applicable treatment for high-, middle, and low-income countries.

In this study, the HepG2 cell line was again chosen for experiments because of its good representation of the patient population with liver cancer treated in our department, where the majority classifies as Caucasian male, and in literature [23]. However, the previously used cell suspension setup, designed in our department, was replaced by classic EP cuvettes. Together with the ESOPE-protocol, of which the RE effectiveness had been previously proven for the HepG2 cell line (Fig. 2), it allowed for better comparison with relevant literature. An effect of pH and temperature changes due to pulsing on cell viability can be excluded in this study. As before, pH changes were minimized by using stainless-steel electrodes and a buffered, low-conductivity solution. In addition, the ESOPE protocol has shown not to significantly change the temperature of the samples. [23]

However, there are some limitations to the used methods. During (almost) complete cell death, some HepG2 cell viability values (ECT with bleomycin) measured by the MTT assay, appeared to be negative. However, there was no significant difference between the positive and negative values, resulting in an overall 0% cell viability. Some values exceeded 100% viability (2.5 µM cisplatin non-electroporated). This specific treatment might have stressed the cells, causing an increase in their metabolism as a response, detected by the MTT assay [33]. In addition, to get an initial idea about the suitable IRE and RE pulse parameters, and the efficacy of ECT and CaEP, the initial study as well as this follow-up study have been performed in vitro using HepG2 cells. As IRE and ECT work in cell suspension as well as in deep seated tumors, in vitro studies for CaEP with hepatic cell lines are an important indicator and basic prerequisite for animal experiments. As limitation, these results are not directly translatable to the clinic, as the in vivo environment is more complex.

To conclude the current study, ECT with bleomycin or cisplatin, or CaEP were more effective in reducing the HepG2 cell viability in vitro using the ESOPE protocol compared to the non-electroporated drug treatments alone. When comparing ECT, HepG2 cells are more sensitive to bleomycin than cisplatin, in similar concentrations. CaEP has the same effectiveness as ECT with bleomycin, but calcium potentially has a better safety profile and several treatment advantages. Despite these promising results, more research is needed to transfer CaEP as novel cancer treatment to the clinic, and to extend the application of traditional ECT to deep-seated tumors like liver cancer.

## Abbreviations

ANOVA: Analysis of variance
ATP: Adenosine triphosphate
CaEP: Calcium electroporation
CAM: Calcein acetoxymethyl ester
ECT: Electrochemotherapy
EMEM: Eagle’s minimum essential medium
EP: Electroporation;
ESOPE: European Standard Operating Procedure on Electrochemotherapy
FBS: Fetal bovine serum
HCC: Hepatocellular carcinoma
IRE: Irreversible electroporation
PEF: Pulsed electric field
PI: Propidium iodine
RE: Reversible electroporation
SEM: Standard error of the mean
